# Experiencias de afrontamiento de la enfermedad en pacientes hospitalizados en Ibagué-Tolima

**DOI:** 10.15446/rsap.V25n3.107220

**Published:** 2023-05-01

**Authors:** Julio E. Mazorco-Salas, Paula K. Carvajal-Hernández, Natalia Villanueva-Mejía

**Affiliations:** 1 JM: Filós. Psicól. M. Sc. Salud Mental Comunitaria. M. Sc. Educación. Universidad de Ibagué. Ibagué, Colombia. julio.mazorco@unibague.edu.co Universidad de Ibagué Universidad de Ibagué Ibagué Colombia julio.mazorco@unibague.edu.co; 2 PC: Psicól. Universidad de Ibagué. Ibagué, Colombia. paulacarvajalh@gmail.com Universidad de Ibagué Universidad de Ibagué Ibagué Colombia; 3 NV: Psicól. Universidad de Ibagué. Ibagué, Colombia. natisvillame@hotmail.com Universidad de Ibagué Universidad de Ibagué Ibagué Colombia

**Keywords:** Adaptación psicológica, continuidad de la atención al paciente, servicio de urgencia en hospital, acontecimientos que cambian la vida *(fuente: DeCS, BIREME)*, Adaptation psychological, continuity of patient care, emergency service, hospital, life change events *(source: MeSH, NLM)*

## Abstract

**Objetivo:**

Se buscó identificar las experiencias de afrontamiento de la enfermedad en pacientes de los servicios de hospitalización en piso y la unidad de cuidados intensivos coronaria de una clínica en Ibagué (Tolima).

**Métodos:**

Con una aproximación cualitativa, se aplicaron dos instrumentos a 15 pacientes masculinos y femeninos, entre las edades de 25 y 78 años que vivenciaron diferentes patologías de carácter crónico.

**Resultados:**

Las experiencias de afrontamiento de los pacientes dan cuenta de la existencia de repertorios de acción y omisión en torno al afrontamiento y el cuidado de la enfermedad, así como prácticas de bienestar. Se explicitaron categorías acerca de las preocupaciones, lo difícil, lo bonito, lo que ayuda a llevar la enfermedad, lo que ayuda al bienestar y las proyecciones de deseo al superar la crisis de hospitalización.

**Discusión:**

La ruptura con las formas de vida es una de las principales dificultades que, sumado a la enfermedad, la incertidumbre y la calidad del servicio hospitalario genera condiciones favorables o no para el afrontamiento.

**Conclusiones:**

Poder identificar las estrategias de afrontamiento de los pacientes dentro del tiempo de hospitalización puede llegar a influir de manera positiva o negativa en su recuperación. Se hace relevante reconocer y dar lugar a los saberes y las prácticas desde la voz de los propios pacientes, para generar acciones coherentes y respetuosas con distintas formas de vivir, cuidar y morir.

## Abstract (en)

**Objective:**

We sought to identify the experiences of coping with illness in patients of the Hospitalization services on the floor and the Coronary Intensive Care Unit of a Clinic in Ibagué (Tolima).

**Materials and Methods:**

With a qualitative approach, 2 instruments were applied to 15 male and female patients, between the ages of 25 and 78 years old, who experienced different chronic pathologies.

**Results:**

The patients' coping experiences show the existence of repertoires of action and omission regarding coping and care of the disease, and wellbeing practices. Categories about worries, what is difficult, what is beautiful, what helps to cope with the disease, what helps to wellbeing and projections of desire when overcoming the hospitalization crisis were made explicit.

**Discussion:**

The rupture with ways of life is one of the main difficulties, which, added to the disease, uncertainty and the quality of the hospital service generates favorable or unfavorable conditions for coping.

**Conclusions:**

Being able to identify the coping strategies of patients within the hospitalization time can have a positive or negative influence on their recovery. It is relevant to recognize and give place to the knowledge and practices from the voice of the patients themselves, in order to generate coherent and respectful actions with different ways of living, caring and dying.

Desde el momento en que una persona es diagnosticada con una enfermedad crítica o su tratamiento necesita un ingreso hospitalario, tanto el paciente como sus familiares o cuidadores primarios se ven inmersos en un proceso complejo, durante el cual pueden experimentar diferentes alteraciones emocionales 1. Es innegable que el hecho de ingresar a una clínica representa para el paciente un evento con un gran impacto físico y emocional, en el que los temores, la soledad y la pérdida de autonomía son condiciones frecuentes que confrontan a los actores de estas unidades con la vulnerabilidad, el sufrimiento y la muerte 1,2.

Durante la hospitalización en la clínica, las afectaciones emocionales citadas con mayor frecuencia por los pacientes son la ansiedad, el estrés, la depresión o el denominado síndrome de cuidados intensivos. Posteriormente al afrontamiento de la enfermedad, las alteraciones emocionales pueden persistir, en ocasiones por varios meses después de haberse dado el alta hospitalaria, llegando a desarrollarse trastornos de estrés postraumático, lo que justifica un seguimiento con el objetivo de detectarlas, tratarlas adecuadamente y prevenirlas 3.

Asimismo, en el contexto pandémico actual producto del covid-19, los pacientes que necesitan una hospitalización más extensa en la UCI tienden a ser más susceptibles a desarrollar problemas o alteraciones en su salud mental como el delirio, trastornos de estrés postraumático, ansiedad y depresión. Además, otros factores como las restricciones en las visitas de sus familiares y la incertidumbre por el diagnóstico o el temor de tener una enfermedad o infección grave o con tratamiento difícil, aumentan en ellos la presencia de estas alteraciones 4.

El afrontamiento y la adaptación son conceptos importantes para la calidad de vida de un individuo porque son fenómenos dinámicos en los cuales se realizan procesos de integración entre el paciente y el entorno, que crean estilos y estrategias para afrontar una situación, en este caso una enfermedad difícil y la estadía en el hospital 5-10.

## MÉTODOS

La presente investigación tiene un diseño cualitativo de tipo descriptivo 11, pretende describir las experiencias de afrontamiento de la enfermedad en un grupo de pacientes con diferentes diagnósticos crónicos y padecimientos físicos. La información se recolectó en un momento único y determinado en el tiempo con la finalidad de describir el modo en el que se presentan las categorías de investigación, por medio de instrumentos de tipo cualitativo.

### Participantes

El muestreo intencionado incorporó a personas ingresadas en los servicios de cuidados coronarios y hospitalización de la clínica. Los participantes tuvieron diagnósticos tales como diabetes mellitus tipo 2, hipertensión arterial, cáncer, insuficiencias renales, enfermedades cardiovasculares, enfermedades gástricas y enfermedades respiratorias. Como criterio de inclusión se tuvo en cuenta que el cuerpo médico y de enfermería solicitaran valoración por psicología, que las personas no estuvieran intubadas, que se encontraran en estado de consciencia, orientadas y con capacidad de expresión oral. El muestreo intencionado final estuvo constituido por un total de 15 pacientes entre hombres y mujeres, con edades entre 25 y 78 años. Tal como se aprecia en la Tabla 1, la distribución de los participantes hombres y mujeres fue de 53,3% y 46,6%, respectivamente.

**Tabla 1:** Características de los participantes (pacientes) según variables estipuladas

	Características	N	%
Sexo	Mujer	8	53,3
	Hombre	7	46,6
Edad	Entre los 15 y 35 años	2	13,3
	Entre los 36 y 55 años	6	40,0
	De 55 a más años	7	46,6
Servicio	Hospitalización	7	46,6
	Unidad de cuidados intensivos coronaria	8	53,3
Tiempo de hospitalización	Entre 1 a 5 días	3	20,0
	Entre 6 a 15 días	4	26,6
	Entre 15 a 30 días	6	40,0
	De 30 a más días	2	13,3

### Instrumentos y análisis

Se utilizaron dos instrumentos: una encuesta de caracterización de los participantes y una entrevista semiestructurada, la primera para conocer el perfil socio-demográfico y la segunda estuvo orientada a indagar en las experiencias de afrontamiento de la enfermedad en los pacientes. Estos instrumentos fueron diseñados por el equipo de investigadores, con base en la literatura en el campo y a través de un proceso de validación de expertos y pilotaje de tales instrumentos. Estos fueron evaluados por tres expertos en campos como la psicología de la salud, la psicometría y la investigación cualitativa. La información fue registrada y trascrita, y se hizo un análisis cualitativo de generación y asociación de categorías emergentes a partir de las respuestas emitidas. El análisis se apoyó en el software de análisis de información Atlas ti 22.

### Consideraciones éticas

De acuerdo con el Código Deontológico y Bioético y la Ley 1090 de 2006, los participantes firmaron un consentimiento informado que contempla las condiciones de autonomía, confidencialidad, beneficencia y no maleficencia de la investigación. Asimismo, se les informó acerca de los alcances, los compromisos y los propósitos investigativos de la aplicación de instrumentos.

## RESULTADOS

### Las experiencias de los pacientes

Los resultados obtenidos del cuestionario para identificar las vivencias de afrontamiento de los pacientes se presentan por secciones en las cuales se plasman las diferentes percepciones, pensamientos y experiencias durante la estancia de los pacientes en la clínica.

A continuación se presentan los resultados a partir de categorías emergentes del análisis cualitativo de la información como: preocupaciones ante la enfermedad, experiencias negativas en el periodo de hospitalización, lo que ayuda a afronta la enfermedad, lo bonito que se experimentó durante la hospitalización, las prácticas de afrontamiento de la enfermedad y las esperanzas y deseos para la recuperación y el alta hospitalaria.

### Preocupaciones

Se identifica que desde el momento en que los pacientes enfermaron, lo más difícil de afrontar y lo que más les ha preocupado han sido los impactos físicos experimentados, como los efectos de la postración, los procedimientos médicos a los cuales se tienen que someter y el proceso de recuperación. Como lo menciona un participante, "es muy difícil tener que estar postrada en esta cama todo el día, sin poder hacer las cosas como ir al baño, moverme, bañarme y que todo me lo hagan las enfermeras" (Participante 12, cuestionario 1, 19 de abril, 2022).

**Figura 1:**
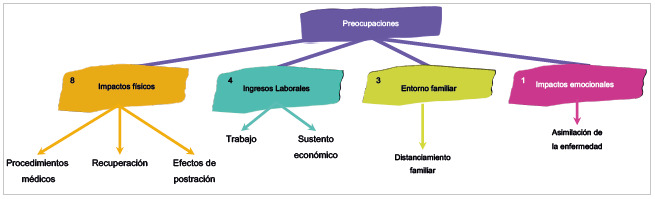
Diagrama de relación de las categorías y las subcategorías del cuestionario entregado a los pacientes: Preocupaciones

Además, como se refleja en la Figura 1, dentro de las categorías mencionadas por los pacientes, los ingresos laborales son un tema que les preocupa; no poder trabajar y por ende no recibir un ingreso económico, el cual se convierte en el sustento diario de muchos de los pacientes. Como lo refiere un participante, "La cuestión de trabajar, no poder conseguirse la plata por toda esta cuestión" (Participante 5, cuestionario 1, 13 de abril, 2022). Y para otro participante: "Que no iba tener yo como trabajar, el asunto económico, eso fue lo más difícil, que me pregunte, ¿y ahora cómo?" (Participante 2, cuestionario 1, 11 de abril, 2022). En seguida se identifican las preocupaciones por el distanciamiento familiar y la asimilación emocional de la enfermedad, como se puede observar en la Figura 1, en la que se ubica la categoría central, las subcategorías con el número de recurrencia y los nodos emergentes.

## Experiencias negativas

**Figura 2:**
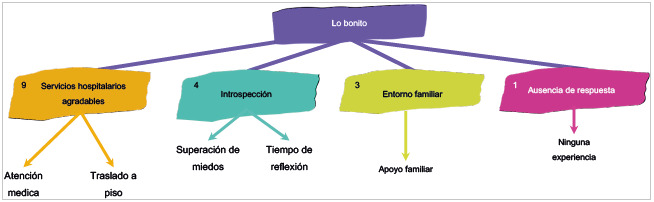
Diagrama de relación de las categorías y las subcategorías del cuestionario entregado a los pacientes: Experiencias negativas

Como se evidencia en la Figura 2, las categorías recurrentes fueron los impactos físicos, los impactos emocionales, la negligencia y la ausencia de respuesta. Sobre los impactos físicos, se reconoce la dificultad en la recuperación lenta y el diagnóstico desconocido como factores que hacen el proceso de afrontamiento más complejo y frustrante. Esto se logró percibir con respuestas como la de una participante que refiere: "pensaba que mi situación era más sencilla y me iba a recuperar más rápido y no fue así" (Participante 14, cuestionario 1, 20 de abril, 2022).

En cuanto a la categoría de impactos emocionales, se comentó acerca del malestar e incluso enojo por no ser escuchados y no poder expresar con libertad sus necesidades, así como asimilar el hecho de estar hospitalizados, ver morir a sus compañeros de habitación y aun, en el caso de una participante, ser acosada sexualmente por un tripulante de ambulancia. Estas experiencias desfavorables alteran la regulación emocional de los pacientes, además de su enfermedad actual; unas de las respuestas de los pacientes referente a esas experiencias negativas fueron: "tenía otro concepto de T diferente, lo que me asustó mucho, no sabía lo que me esperaba ahí, fue una experiencia traumante para mi" (Participante 10, cuestionario 1, 19 de abril, 2022); "ayer se murió el chico que estaba en la otra cama, pero eso fue horrible, fue como si se me hubiera muerto un hermano, no le deseo esa experiencia a nadie" (Participante 9, cuestionario 1, 19 de abril, 2022).

Además, se presenta la categoría de negligencia, vinculada a las experiencias que los pacientes tuvieron, como la tardanza en valoraciones y complicaciones en trámites, que tienen repercusiones en la recuperación, el tratamiento y el estado emocional. Por último, se encuentra la categoría de ausencia de respuesta, puesto que tres de los pacientes no consideran haber vivenciado una experiencia negativa durante su estancia.

### Lo que no ayuda

**Figura 3:**
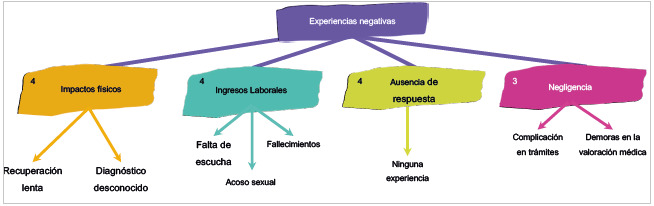
Diagrama de relación de las categorías y las subcategorías del cuestionario entregado a los pacientes: Lo que no ayuda

Como se muestra en la Figura 3, las principales vivencias que no ayudan a sentirse bien se relacionan con la pérdida de autonomía. Esto se debe al cambio de hábitos, los impactos físicos y la dependencia del personal médico. Los participantes lo refieren así: "el hecho de no poder hacer lo mismo de antes" (Participante 12, cuestionario 1, 19 de abril, 2022), "no poder moverme y tener que hacer en un pato o pañal" (Participante 10, cuestionario 1, 19 de abril, 2022); o efectos de la hospitalización y procedimientos médicos: "sentirme cansado y sin vitalidad" (Participante 6, cuestionario 1, 20 de abril, 2022); "me duele la cola y la espalda de estar en esta cama" (Participante 3, cuestionario 1, 11 de abril, 2022); "tener que hacerme la diálisis día de por medio" (Participante 5, cuestionario 1, 13 de abril, 2022). Estas condiciones no favorecen que el paciente pueda experimentar una hospitalización tranquila y de reposo.

Aunque son situaciones inevitables, se convierten en cambios repentinos, incómodos y duros de aceptar, especialmente para aquellos participantes con edades entre los 50 y los 60 años.

La ausencia familiar y la falta de acompañamiento en los servicios de hospitalización no contribuyen a sentirse bien en el proceso de enfermedad. Por el lado de la unidad de cuidados intensivos coronaria, la visita de tan solo 30 minutos se convierte en algo difícil de aceptar por los pacientes, puesto que antes de su enfermedad compartían la mayoría de su tiempo con sus familiares. Como lo expresa un participante, "extraño mucho a mi familia, es duro no poderlos ver casi, en especial a mi hija" (Participante 13, cuestionario 1, 19 de abril, 2022); "eso me genera mucha soledad" (Participante 13, cuestionario 1, 19 de abril, 2022). Y por el lado de hospitalización en piso, muchos de los familiares no pueden quedarse las 12 de horas completas acompañándolos, puesto que deben trabajar o cumplir con otras responsabilidades, por lo cual los pacientes experimentan soledad.

Adicionalmente a las categorías antes mencionadas, se encuentran las categorías de pensamientos negativos, como pensamientos de miedo, duda y preocupación, tales como "tengo pensamientos negativos como el miedo de no salir de esta o saber cómo voy a quedar después de esto" (Participante 1, cuestionario 1, 11 de abril, 2022), o "yo misma no me ayudo a sentirme bien, porque mantengo pensando en cosas malas o preocupada" (Participante 4, cuestionario 1, 13 de abril, 2022). Además de eso, se suman impactos emocionales como episodios de estrés o disgusto por la comida que reciben el hospital.

### Lo bonito

Más de la mitad de los pacientes refirieron que los servicios hospitalarios fueron agradables. Ello se convirtió en una vivencia significativa puesto que la colaboración y la buena atención por parte del personal se convierten en factores positivos para lograr afrontar la estancia en hospitalización y el proceso de enfermedad que viven los pacientes. Esto se evidencia en la voz de un participante: "la gente se porta bien con uno, vienen a cambiarle el líquido, a hacerme visita y qué más bueno que lo traten a uno bien, se siente uno más en confianza" (Participante 13, cuestionario 1, 19 de abril, 2022).

**Figura 4:**
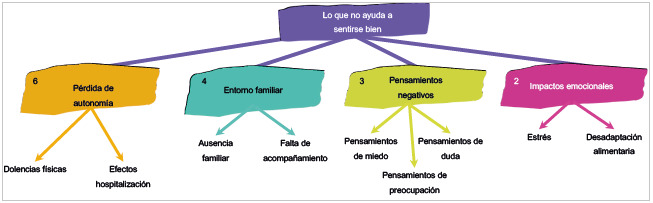
Diagrama de relación de las categorías y las subcategorías del cuestionario entregado a los pacientes: Lo bonito

La introspección se hace significativa en la experiencia, así como los momentos de reflexión y superación de miedos, como lo mencionan los participantes: "aprender a llevar la vida con calma" (Participante 5, cuestionario 1, 13 de abril, 2022); "he tenido tiempo para hablar con Dios, reflexionar y solucionar algunos problemas personales" (Participante 12, cuestionario 1, 19 de abril, 2022). Y el apoyo y la cercanía familiar se convierten en una estrategia para asimilar y manejar los cambios y las afectaciones que la misma enfermedad y la estancia producen: "encontrarse y hablar con familiares con quienes no tenía contacto hace mucho además sentir la unión y el amor de todos en este momento" (Participante 9, cuestionario 1, 19 de abril, 2022). A su vez, hubo un paciente, como se muestra en la Figura 4, que expresó que no le pasó nada "bonito" o "fácil" desde el momento en que se enfermó.

### Prácticas de afrontamiento

**Figura 5:**
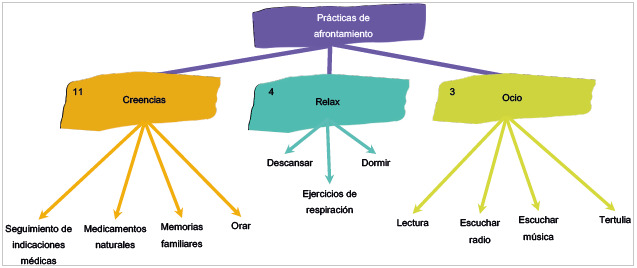
Diagrama de relación de las categorías y las subcategorías del cuestionario entregado a los pacientes: Prácticas de afrontamiento

En la Figura 5 se explicitan las categorías en las que 11 pacientes manifestaron que sus creencias les ayudan a hacer la enfermedad más llevadera. Por ello, en estos casos se hace importante orar, hablar con los familiares, tomar medicamentos naturales como aguas de hierbas y otros rituales con estas, además de seguir las indicaciones del médico. Estas son estrategias personales centradas en el saber de la vida cotidiana que adoptan cada uno de los pacientes: "Tomar medicamentos naturales, muchas aguas de hierba" (Participante 7, cuestionario 1, 20 de abril, 2022).

Por otro lado, cuatro de los pacientes encuestados se identificaron con la categoría relax, donde se ubica el tiempo para descansar, dormir, y hacer ejercicios de respiración para distraer la mente de la situación actual. Como lo menciona un participante, es importante "concentrarse en la respiración, para no pensar en la enfermedad" (Participante 4, cuestionario 1, 13 de abril, 2022). Por último, se encuentra la categoría de ocio, en la cual tres pacientes señalaron actividades como leer, escuchar radio, tertuliar y escuchar música como aquellas que suelen hacer en su rutina diaria, y seguir realizándolas durante su hospitalización los hace sentir activos y aliviados. De esta manera lo refiere un participante: "leer y escuchar música me hace sentir alegre y funcional" (Participante 11, cuestionario 1, 19 de abril, 2022).

### Las esperanzas y los deseos

**Figura 6:**
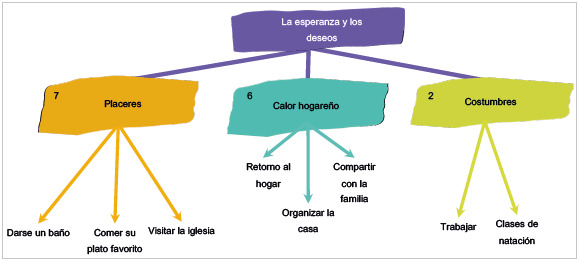
Diagrama de relación de las categorías y las subcategorías del cuestionario entregado a los pacientes: La esperanza y los deseos

En la Figura 6 se representa, en primera instancia, la categoría de placeres, que fue la que se mencionó con mayor recurrencia. Comer su plato favorito, darse un baño y visitar la iglesia son acciones que anhelan realizar de manera inmediata en el momento que les den egreso, ya que son actividades que por las indicaciones que deben seguir durante la hospitalización no pueden hacer, aunque les generan gran satisfacción. Cuando los pacientes se refieren a comidas como chicharrón, sudado de bagre, gallina criolla, pasta con carne de cerdo y patacón, se ve en sus rostros el deseo y la felicidad que el saborear esos platos les depararía. Una ducha se convierte en una de sus mayores aspiraciones, como lo expresó con gran ilusión un participante: "ya quiero llegar a mi finca, que tiene un patio grande y darme un baño de agua fría muy rica al sol y bañarme como no he podido todo este tiempo, eso es lo primero que quiero hacer cuando salga de acá" (Participante 10, cuestionario 1, 19 de abril, 2022).

Otra categoría central para el deseo de mejoría es el calor hogareño: retornar al hogar, organizar la casa y compartir en familia son situaciones que los pacientes no ven la hora de vivenciar de nuevo. Así, manifiestan al respecto: "poder volver a estar con mis hijos" (Participante 7, cuestionario 1, 20 de abril, 2022) ; "irme para la casa y estar con mi niña" (Participante 13, cuestionario 1, 19 de abril, 2022); "irme para la casa y hablar otra vez con mi señora" (Participante 3, cuestionario 1, 11 de abril, 2022); e "irme para Neiva y poder reencontrarme con mi familia" (Participante 6, cuestionario 1, 20 de abril, 2022). Lo anterior muestra la importancia del vínculo familiar, la red de apoyo y la unión familiar para el afrontamiento.

La categoría costumbres también se vio reflejada en el relato de los pacientes, donde a algunos de ellos lo primero que se les antoja hacer cuando salgan de la clínica es trabajar e ir a clases de natación, retornar a sus responsabilidades, estilos de vida, rutinas e incluso hobbies y actividades de entretenimiento, lo que para ellos se convierte en algo placentero y se asocia con la recuperación de la salud y la superación de la enfermedad. Como lo menciona un participante, “quiero irme a trabajar de nuevo, camellar como antes de estar aquí, moverme como antes y sentirme con más vitalidad” (Participante 1, cuestionario 1, 11 de abril, 2022).

## DISCUSIÓN

En los resultados obtenidos en esta investigación se pudo evidenciar que el afrontamiento es una categoría emergente, producto de la sumatoria de los esfuerzos individuales, familiares y del personal médico para sobrellevar la enfermedad, los cambios que esta trae y adaptarse a ella, como lo menciona McHaffie 12. Al recolectar las respuestas del cuestionario aplicado, se demostró cómo el paciente se encuentra en una búsqueda constante de acciones a lo largo de su estancia que le permitan sentir que puede tolerar la enfermedad y la hospitalización y adaptarse a los cambios con los que se encuentra, así como proyectar un futuro mejor.

De acuerdo con Cassaretto et al. 13, durante la etapa de hospitalización los pacientes experimentan un proceso de afrontamiento de las situaciones estresantes y los síntomas de la enfermedad. Entre tales situaciones estresantes se encuentran la falta de conocimiento y la incertidumbre sobre la unidad de cuidados intensivos (UCI), su funcionamiento, el periodo de permanencia, la gravedad del padecimiento y el bajo nivel de información y comunicación, lo cual interfiere en el proceso de afrontamiento de la enfermedad durante la hospitalización. El participante 10 sostuvo al respecto: "tenía otro concepto de UCI diferente, lo que me asustó mucho, no sabía lo que me esperaba ahí, fue una experiencia traumante para mí". Ello género en este la implementación de estrategias perjudiciales para su bienestar al inicio de su estancia y la identificación de aquello que hizo desencadenar una actitud de afrontamiento.

Otra característica que se percibió de manera notoria en la investigación fue la perturbación que produce en los pacientes el ingresar a un servicio de hospitalización, como impactos físicos y emocionales, preocupaciones por los ingresos laborales y distancia con el entorno familiar 14-19. Estos autores vinculan el proceso de ruptura de la vida cotidiana, por cuanto el paciente es "arrancado" de su casa, de su familia y de su vida. Al ingresar a hospitalización, se percibe por parte de los pacientes una sensación de desamparo físico, mental y emocional, así como sensaciones de aislamiento del entorno, contexto y hábitos de la vida cotidiana.

Un dato que contrastar citado en los antecedentes de la investigación es que "se percibe que las mujeres tienen un mayor repertorio con respecto a estrategias de afrontamiento, pero menos habilidad que los hombres para enfrentar los problemas" 20. Prácticas como la lectura, tertuliar, orar y escuchar música fueron mencionadas en más ocasiones por pacientes del género femenino, lo que muestra una gran variedad de estrategias adoptadas por mujeres para afrontar la enfermedad.

De conformidad con los resultados obtenidos en esta investigación, los tipos de estrategias de afrontamiento más utilizadas por los participantes son las estrategias de afrontamiento enfocadas en el problema y aquellas orientadas a la regulación emocional. El primer tipo de estrategias de afrontamiento se reconoció en los resultados, debido a que los pacientes expresaron llevar a cabo acciones que definieron y formularon el problema, en las que no se consideró la enfermedad como algo insuperable. Esto se ve reflejado en expresiones de los participantes como "tengo el pensamiento de que si supero esta situación, nada me va a detener de ahora en adelante" (Participante 4), o "aprender a llevar la vida con calma y poder irme recuperando" (Participante 5), las cuales estuvieron implícitas en experiencias positivas o lo más bonito que les pasó durante la instancia, reconociendo así su situación compleja como algo habitual y normal de la vida, que puede presentarse en cualquier momento.

Además, los pacientes tienden a buscar alternativas o soluciones para afrontar su enfermedad con el fin de minimizar los efectos negativos que estar sometidos a una hospitalización trae consigo. Algunas de esas acciones, como se aprecia en la Figura 5, son tomar medicamentos naturales, seguir las indicaciones médicas al pie de la letra, e incluso orar, todo según el sistema de creencias que cada paciente tiene. Esto genera en ellos también una toma de decisiones que les permite buscar la recuperación, como por ejemplo la aprobación de procedimientos y tratamientos con el fin de encontrar un bienestar tanto físico como emocional.

Por otro lado, el segundo tipo de estrategia de afrontamiento se vio evidenciado con gran frecuencia en los participantes en categorías como ocio, relax, creencias, placeres, calor hogareño y costumbres, plasmadas en las figuras 5 y 6, puesto que son una gran variedad de acciones que permiten a los pacientes reducir y manejar el malestar emocional asociado a su situación de enfermedad y hospitalización, que aun cuando en cierta medida no resuelven en su totalidad el problema o situación compleja, sí minimizan algunas reacciones, lo que genera una sensación de mejoría y afrontamiento. Así, este tipo de estrategias producen un cambio en la forma de interpretar el problema y mantener un equilibrio en los pensamientos que se generan al respecto.

Por consiguiente, se puede ver en los resultados la presencia de dos de las tres estrategias de afrontamiento establecidas por Lazarus et al. 21, así como respaldar la idea de que en realidad existen muchas estrategias posibles de afrontamiento que pueden utilizar un individuo y su entorno. El uso de unas u otras en la mayoría de los casos suele estar determinado por la naturaleza del estresor y las circunstancias en las que se produce 22.

Por último, entre los aspectos que se consideran de gran importancia para el afrontamiento, de acuerdo con las categorías establecidas por las respuestas de los pacientes, se encuentra el papel de la humanización del entorno y el rol del profesional, en cuanto al apoyo que ofrece a la familia y al paciente para generar estrategias de afronta-miento que permitan promover la atención integral en los servicios de salud y brindar cuidado de calidad 5.

Tras el análisis de los resultados obtenidos es posible concluir que todos los pacientes que atraviesan por un proceso de hospitalización viven diferentes experiencias, tanto positivas como negativas, con emociones constantes como preocupación, miedo y frustración. Al inicio de su proceso, esto conlleva reacciones de rechazo a la enfermedad, temor constante a no recuperarse exitosamente y no reincorporarse al estilo de vida que se llevaba antes de la enfermedad. Igualmente, el impacto que tiene en ellos el deterioro de las relaciones sociales y familiares, como también de su situación económica, entre otros factores, es muy grande e influye en su forma de percibir su situación y la necesidad de tomar medidas.

Además, se identificaron las estrategias de afronetamiento que adoptan los pacientes durante la hospitalización, teniendo en cuenta que se encuentran en un contexto vulnerabilizado, donde su estilo de vida se transforma, lo que genera rupturas con la vida cotidiana y un entorno de incertidumbre que supone cambios en la vivencia emocional y su regulación. Estas condiciones constituyen barreras para el afrontamiento y aumentan las defensas emocionales, así como los estados de irritabilidad, tristeza e impotencia y los deseos de abandonar el tratamiento. Todas estas reacciones se asocian con el proceso de recuperación. Este panorama no solo lleva a considerar la relevancia de tratar la enfermedad en su carácter físico y biológico, sino que demanda el desarrollo de condiciones para la salud, el cuidado emocional y el bienestar.

A partir de los resultados se logra visualizar que existe una gran diversidad de respuestas por parte de los pacientes, en las cuales se evidencia que ya tienen estructurado un concepto de afrontamiento y con ello algunas estrategias que han percibido que les funcionan para hacer que su estadía en la clínica sea más llevadera. Es importante para ellos el hecho de sentirse útiles y activos, aun si se encuentran postrados en una cama. Por lo tanto, realizar actividades como leer, pintar, escuchar música, entre otros, ayuda a distraer su mente del contexto donde están y canalizar emociones fuertes. Esto genera en los pacientes una reducción de los estresores fisiológicos y psicosociales a niveles tolerables para adaptarse a la nueva situación.

Finalmente, se recomienda capacitar al personal de salud en cuanto a pautas para brindar un servicio integral donde se proporcione un ambiente de confianza, amabilidad y calidad en salud que contribuya a su proceso de afrontamiento, recuperación y bienestar, tanto para los pacientes como para sus familias y entornos. De igual manera, se problematiza la necesidad de incorporar al equipo de salud un mayor número de profesionales de psicología, trabajo social y saberes asociados al cuidado. De igual manera, se resalta la necesidad de reconocer y dar lugar a los saberes y las prácticas desde la voz de los propios pacientes, tanto como del personal de salud, como ruta de generación de acciones coherentes y respetuosas con distintas formas de vivir, cuidar y morir ♣
